# Stoichiometry and the New Biology: The Future Is Now

**DOI:** 10.1371/journal.pbio.0050181

**Published:** 2007-07-17

**Authors:** James J Elser, Andrew Hamilton

**Affiliations:** Princeton University, United States of America

## Abstract

There is a call for biological science to move away from the reductionist focus of the past, but there are large-scale integrative efforts already underway; biological stoichiometry provides one such example.

## The Future Is Now

The world is an untidy place, and the sciences—all of them—reflect this. One source of this untidiness is the relationship between levels of organization. Reducing macrolevels to microlevels—explaining the former in terms of the latter—has met with successes but has never been the whole story. In the biological sciences, there has been much attention lately to the shortcomings of reductionism on the grounds that (i) it changes the subject rather than explaining, (ii) it leads to a myopically molecular view of the biological world, and (iii) the behavior or behaviors of complex systems are often very poorly predicted based solely on their microproperties. It is just for these reasons that biologists of many stripes have called for a move away from reductionism and toward a new kind of biology for the 21st century. But what shape might this new biology take?

A look at the recent literature reveals that the new biology should be mathematical in nature, partly because of needed improvements in modeling the outcomes of interactions between levels of organization [1]. The new biology should be integrative across levels of organization while remaining attentive to differences in kinds and degrees of causal factors [2]. It should also attend to the holistic, nonlinear, and emergent features of the biological world, focusing primarily on “evolution and the nature of biological form” [3]. The new biology should also move “away from a reductionist focus on a limited number of molecular components to a comprehensive understanding of how large numbers of interrelated components of a system comprise modules or networks whose functional properties emerge as definable phenotypes” (http://www.systemsbiology.org). Its approaches “should be more integral, multilevel, and dynamic” than they are presently [4]. It should also offer new theoretical insights that help make sense of and integrate the vast amounts of data being produced by biologists in many fields [5].

Most of these calls for the wholesale rethinking of biological science are motivated by the successes and failures of molecular biology in the second half of the 20th century. There are already, however, large-scale integrative efforts underway outside of molecular biology that are not reductive, or at least are not reductive in the way that has concerned those who worry about an undue focus on molecules. Recent work on metabolic scaling theory [6) comes to mind as an example, as does the recent emphasis on mechanisms in context in systems biology [7]. These efforts are interlevel, quantitative, attend to nonlinear and emergent features, aim at being comprehensive, and give attention to evolutionary constraints on form and function. Here we explore a third example, biological stoichiometry, in detail, and argue that what biology needs is not so much a new epistemology, method, or vision, but to notice that integration of the kind called for by those who are dissatisfied with the myopia of molecular biology is already a focus within several fields of biological inquiry. The new biology is already here.

## Integration in Ecological and Biological Stoichiometry

Stoichiometry is the application of laws of matter conservation and of definite proportions to the understanding of the rates and yields of chemical reactions given a set of reactants. Ecological stoichiometry recognizes that organisms themselves are outcomes of chemical reactions and thus their growth and reproduction can be constrained by supplies of key chemical elements [especially carbon (C), nitrogen (N), and phosphorus (P)] [8]. Much stoichiometric work lies in the characterization of the elemental composition of organisms and in understanding how closely their chemical composition is regulated (“stoichiometric homeostasis”), and thus the extent to which their growth conforms to a law of definite proportions.

Whereas breaking organisms and ecosystems down into their elemental compositions is reductive in nature, ecological stoichiometry does not stop there. Take, for example, the application of stoichiometry to explain observations in freshwater ecology showing that changes in food-web structure can affect the relative availabilities of the key limiting nutrients N and P in lakes [9]. These changes result from cascading effects of food-web structure, which alter the relative abundance of herbivorous zooplankton species in the community [10]. Specifically, lakes with four dominant trophic levels (phytoplankton, zooplankton, planktivorous fish, and piscivorous fish) are generally dominated by the large-bodied and P-rich (low C: P, low N:P) crustacean Daphnia [11], whereas lakes with three dominant trophic levels (lacking in piscivores) are dominated by low P (high C:P, high N:P) copepods. Thus, alterations in food-web structure cause the zooplankton communities to sequester and recycle N and P differentially [12], in turn affecting the nutrient regime experienced by the phytoplankton community.

Thus, ecological stoichiometry provides an understanding of how food-web structure can affect phytoplankton nutrient limitation due to shifts among dominant zooplankton that differ in C:N:P ratios. But at this point, it is only natural to ask why C:N: P ratios among zooplankton species are so different. Elser and colleagues [13] proposed the “growth rate hypothesis” (GRH) to answer this question, in a step toward a broader theory of biological, rather than ecological, stoichiometry. Specifically, they proposed a growth-rate dependence of C:P and N:P ratios in living things, because organisms must increase their allocation to P-rich ribosomal RNA in order to meet the elevated protein synthesis demands of rapid growth. Support for the GRH in zooplankton soon appeared [14,15]. The GRH not only explains elemental composition in terms of its biochemical basis, but it also provides a clear evolutionary connection (as some advocates of a new biology have urged): evolutionary changes in organismal growth or development rate have physiological and ecological ramifications due to the changes they induce in organismal elemental demands. Evolutionary change requires a genetic mechanism, so Elser and colleagues [16] proposed that selection for changes in growth or developmental rate operates on available genetic variation in transcriptional capacity of the genes that encode for ribosomal RNA, the rDNA. Preliminary support for such mechanisms in Daphnia has been produced [17,18].

While a satisfying reductionist account seems in hand, the effort has opened up multiple avenues for broad integration in which connections are made not by further digging for lower-level mechanisms, but by seeking new connections of two kinds. One kind of connection is horizontal—the aim is to extend the results of reductionistic digging to include other taxa and systems at roughly the same level of organization. Vertical connections, by contrast, attempt to “resurface” by applying the results of mechanistic explanation in one field to make and test predictions about yet-undocumented phenomena at higher levels and in other fields.

In ecological stoichiometry, horizontal integration has been attempted by applying stoichiometric analysis to trophic interactions beyond lakes and freshwater zooplankton. Stoichiometric analysis is readily used for cross-ecosystem comparisons, as in comparison of the stoichiometric structure of lake and marine food webs [19] and lake and terrestrial food webs [20]. Likewise, data were soon produced demonstrating a key role of P-based stoichiometric imbalance in affecting the growth of terrestrial insects [21,22], as had been shown previously for zooplankton [23,24]. Furthermore, the GRH should apply to a variety of biota, not merely freshwater zooplankton. Elser and colleagues [25] showed that zooplankton, bacteria, fruit flies, and other insects display similar growth-RNA-P relationships, whereas Weider and colleagues [26] presented evidence that the functional significance of rDNA variation in explaining such relations is broadly similar across diverse taxa, which are examples of horizontal integration within biological stoichiometry.

Vertical integration has worked somewhat differently in biological stoichiometry. Take, for example, the connections made by applying the GRH to the study of cancer [27]. Elser and colleagues noted that many well-known oncogenes influence the expression of ribosomal RNA genes, increasing production of ribosomal RNA. This suggests that rapidly growing tumor tissues may have unusually high P demands and thus may experience P-limited growth. While clinical data suggest that proliferating tumors can deplete body P supplies, testing these ideas with existing information has proven difficult. New efforts are underway to compare the C:N: P stoichiometry of tumor and normal tissues directly. These confirm that colon and lung tumors are indeed more P-rich than normal tissues (JJ Elser et al., unpublished data), information that can be incorporated into simulation models to assess whether such differences might affect tumor progression [28].

Vertical integration works here by thinking mechanistically rather than directly in evolutionary terms: new relationships at higher levels are predicted based on known lower-level mechanisms. In the cancer example, these higher-level phenomena occur in areas of biology that are well outside the scope of the initial investigation. An important feature of the upward integration move made in this case is that it poses questions that may never have been asked at the higher level. Whether or not tumor tissue growth is P-limited only becomes an issue if one has reason to believe that growth rate and P requirements are connected.

We should note that seeking integrative connections—particularly upward across levels of organization from mechanisms identified in other taxa—is unlikely to proceed as cleanly as the reductionist part of the explanatory process in most cases. This is partly because the strength and number of causal factors for different systems vary, even though all systems must also be constrained by the same fundamental thermodynamic rules. For example, the ability of the GRH to explain animal C:P and N: P ratios diminishes with increasing body size, because growth rate scales negatively with size [29]; variation instead is driven by allocation to P-rich bones, with subsequent connections to nutrient cycling processes driven by vertebrates [30]. We think that this relative lack of precision is a feature, rather than a flaw, of upward integration, because it calls attention to opportunities to identify the unique level-, taxon-, or system-specific causal processes that are operational in any particular context.

## Conclusion: New Relations, New Theories

We have argued that the beginnings of a new biology as invoked in the recent literature are already here and we proposed biological stoichiometry as an example. This means only that calls for a new biology are misplaced, not that there is no reason to think carefully about where biology goes next. For example, can stoichiometric theory itself be integrated with other “new biology” efforts that are now emerging, such as systems biology [31] and metabolic scaling theory [29,32]? Our concern for theoretical integration is motivated by a growing problem: biologists have to deal with exponentially expanding data streams from sequencing, profiling, and other high-throughput techniques (e.g., [33]) without the benefit of a device for high-throughput hypothesis generation and testing. It seems that the only hope for creatively interrogating new data is to develop new, integrated theoretical frameworks to inform strategies for that interrogation.

In addition to these broader conceptual tasks, there are questions specific to the stoichiometric realm to be asked and answered. For example: (1) Do observed differences in coupling of P, RNA, and growth rate among species [25] reflect the fact that key aspects of ribosome function (e.g., protein synthesis rate per ribosome) differ considerably among species? This is a question about horizontal integration at the level of comparative functional biology. (2) Can approaches of ecological stoichiometry, largely worked out in studies of competition and producer–consumer relations, be transferred to different kinds of species interactions, such as mutualism or infectious disease [34]? This involves horizontal integration at the level of community ecology. (3) Can the insights of ecological/biological stoichiometry be assimilated into evolutionary theory to better connect disparate evolutionary and ecosystem perspectives [5]? This is a question about vertical integration to link core aspects of cellular biology (e.g., ribosome biogenesis) to large-scale biogeochemistry [35] within adaptive systems and may represent an opportunity for a second “modern evolutionary synthesis.”

Whatever the answers to these or similar questions, the message is clear: integrative, quantitative, causally nuanced, and evolutionarily defensible approaches are currently available for use by biologists. The issue is not that we must wait for a future biology to arrive, but that we should notice and take good stock of what is already underway.
